# Associations among physical activity, diet, non-lifestyle characteristics and the gut microbiome of cancer patients: A scoping review and network analysis

**DOI:** 10.18632/oncoscience.651

**Published:** 2026-03-11

**Authors:** Jerry Armah, Sarah Alzahid, Qinglin Pei, Lakeshia Cousin, Dany Fanfan, Coy Heldermon, Debra Lyon

**Affiliations:** ^1^College of Nursing, University of Florida, Gainesville, FL 32603, USA; ^2^Tampa General Hospital, Nursing Administration, Tampa, FL 33606, USA; ^3^Department of Medicine, University of Florida, Gainesville, FL 32611, USA

**Keywords:** physical activity, diet, gut microbiome, cancer patients, non-lifestyle factors

## Abstract

Lifestyle factors, such as physical activity and dietary modifications can beneficially modulate the gut microbiome of cancer patients, however their effects are often shaped by non-modifiable variables. This review and network analysis aims to synthesize current evidence on how both lifestyle and non-lifestyle factors affect the gut microbiome in cancer patients. A systematic search was conducted on Scopus, CINAHL, PubMed and Web of Science to produce 51 eligible studies for this review. A chi-square test of independence indicated that the distribution of gut bacteria function categories was significantly associated with the category of influencing factor (Χ^2^ = 390.87, *p* = 0.032). Across studies, high physical activity and healthy diets were associated with increased abundances of saccharolytic/short-chain fatty acids and lactic acid-producing bacteria, alongside decreased abundances of pathogenic or opportunistic bacteria. However, these associations may also be influenced by non-lifestyle characteristics such as chemotherapy, age, and cancer type or stage which could mask the benefits of lifestyle interventions. This study highlights the limited but growing evidence linking physical activity, diet and the gut microbiome in cancer populations. Progress in this field will require larger, more integrative designs that account for non-lifestyle confounders and apply advanced analytical approaches to capture complex interactions.

## INTRODUCTION

Cancer survivorship is a distinct phase within the cancer care continuum that extends beyond the completion of treatment that encompasses the long-term physical, psychological, and social challenges that many patients face [1, 2]. Advances in early detection and treatment have contributed to improved survival rates [3, 4]. However, cancer care plans should extend beyond the monitoring of cancer recurrence to address the persistent effects of therapy such as fatigue, metabolic dysfunction, and cognitive decline. Within this evolving landscape of cancer care, the gut microbiome has gained increasing attention as a key mediator of health outcomes among cancer patients [5]. It comprises trillions of microorganisms that live in the gastrointestinal tract, and influence systemic processes such as inflammation, immune response, and neuroendocrine signaling, which are all linked to cancer survivorship in a complex manner. For example, a bidirectional communication pathway between the gut and central nervous system (gut-brain axis) has been linked to neurocognitive and psychological outcomes [6]. Dysregulation of this axis has been implicated in the development of mood disorders such as anxiety and depression, as well as cognitive impairments often referred to as “chemo brain” [7, 8]. Beyond the gut-brain axis, several other physiologic axes link the gut microbiome to cancer risk, progression, and therapeutic response. The gut–immune axis is extensively studied because microbial communities shape both innate and adaptive immunity, which influences chronic inflammation, immune surveillance, and the tumor microenvironment [9–11]. The gut–liver axis is also critical, given the liver’s role in filtering gut-derived metabolites and microbial products; disruptions in this pathway can promote hepatic inflammation, genotoxic stress, and metabolic dysfunction that predispose to malignancy [12–15]. Also, the gut–metabolic/endocrine axis contributes to host metabolism, hormone signaling, and cell proliferation through microbially derived metabolites, such as short-chain fatty acids (SCFAs), bile acids, and tryptophan derivatives [16–18]. These interconnected axes illustrate how the gut microbiome exerts multi-organ influence and creates a complex biological network that influences cancer depending on microbial composition and host context. Given the broad physiological reach of the gut microbiome in cancer survivorship, it may represent both a marker of underlying dysfunction and a modifiable target for cancer intervention.

Lifestyle factors, particularly diet and physical activity (PA), have emerged as significant modulators of the gut microbiome. These factors have implications for both general health and disease-specific outcomes. Several studies have shown that dietary intake directly shapes the composition and metabolic activity of gut microbial communities [19]. For example,

Mediterranean and plant-based diets are high in dietary fiber and polyphenols which have been associated with increased microbial diversity and a higher prevalence of Bifidobacterium and Lactobacillus species [20, 21]. These bacteria produce SCFAs which have been shown to reinforce the integrity of the intestinal barrier, reduce inflammation, and support immune homeostasis [22]. Similarly, PA has been independently associated with alterations in gut microbiota composition, irrespective of dietary intake [23–27]. According to the World Health Organization (WHO), “*Physical activity is any bodily movement produced by skeletal muscles that results in energy expenditure, and exercise is a planned, structured, and repetitive subset of physical activity undertaken to improve or maintain physical fitness*” [28]. Not all physical activity is exercise, however, the WHO recommends the engagement in some forms physical activity with the goal of reducing risk factors for non-communicable diseases [28]. Both animal and human studies have shown that regular moderate-to-vigorous exercise can enhance microbial richness and create a conducive environment for the proliferation of SCFA-producing bacteria, particularly *Faecalibacterium prausnitzii* and *Akkermansia muciniphila* [29, 30]. These microbes have also been linked to anti-inflammatory effects and improved metabolic health [31]. The PA-induced gut microbial shifts occur through several interconnected mechanisms. For instance, exercise reduces gut motility and decreases transit time which creates a lower luminal pH and reduced oxygen tension that favor anaerobic SCFA-producing taxa [32–35]. Also, it exerts antiinflammatory and immunomodulatory effects by reducing pro-inflammatory cytokines and enhancing secretory IgA, which create a microbial niche that supports beneficial commensals [31, 36, 37].

Overall, shifts in the gut microbiome engendered by lifestyle factors, have the potential to strengthen immune function, lower oxidative stress, and improve insulin sensitivity – all of which are physiological changes beneficial for cancer patients [38, 39]. However, these microbiome-mediated benefits are highly context-dependent, and both geography and religion/cultural practices modulate their magnitude. Large cross-country analyses demonstrate that geography is one of the strongest determinants of gut microbiota structure, often explaining more variance than BMI or age, with differences in diversity and dominant taxa between, for example, rural high-fiber, traditional diets and urbanized Western diets [40–42]. Populations consuming minimally processed, fiber-rich, fermented foods generally harbor more SCFA producers and higher diversity compared to Westernized, low-fiber, high-fat patterns which are associated with reduced diversity and more pro-inflammatory profiles [42, 43]. Hence, the same lifestyle interventions may yield different microbiome shifts, and thus different magnitudes of benefit, depending on baseline community structure in each country. Also, culture and religion further add layers to this by shaping what, when and how people eat. For example, religious fasting traditions such as Ramadan, remodel the gut microbiota (increasing alpha diversity and enriching beneficial taxa), even when total calorie intake is not dramatically altered [44–46]. Similarly, cultural norms around vegetarianism, avoidance of specific animal products, alcohol, or particular cooking/fermentation practices produce cultural-specific microbiome signatures [47, 48]. In practice, this means that the potential of beneficial microbiome shifts in cancer patients may not be uniform globally, but is moderated by baseline microbiota shaped by geography, religion and culture.

Beyond modifiable lifestyle behaviors, a range of physiological and clinical factors, many of which are non-modifiable or treatment-related also play a role in shaping the gut microbiome among cancer patients [5, 49, 50]. Chief among these are cancer therapies including chemotherapy, radiation therapy, immunotherapy, and other targeted biological agents. These treatments often have unintended secondary effects on the gut microbiome [51]. Chemotherapy, for instance, has been reported to reduce microbial diversity and alter the relative abundance of key bacterial taxa, which results in a microbial imbalance known as dysbiosis [52, 53]. This treatment-induced dysbiosis has been associated with a range of negative health outcomes that extend into the survivorship phase. For example, decreases in butyrate-producing bacteria following chemotherapy have been associated with greater susceptibility to Clostridioides difficile infection, mucositis, and chronic gut inflammation [54–56]. These gut microbial changes induced by chemotherapy may be lasting, with some studies observing changes 6 months post treatment [57–59]. However, what uniquely defines gut microbial recovery and non-recovery post chemotherapy is still unknown [54]. In addition to treatment exposures, intrinsic host characteristics further influence the gut microbiome’s composition. Physiological factors such as age, sex, and hormonal status alter microbial ecology through mechanisms that are poorly understood [60, 61]. Estrogen has been shown to interact with the gut microbiota through the enterohepatic circulation, with implications for both immune regulation and microbial composition [62, 63]. These host-specific variables can influence the degree to which lifestyle modifications such as physical activity and dietary modifications impact the gut microbiome and may partially explain why some cancer patients do not respond similarly to these lifestyle modifications.

Taken these together, it is evident that there is a complex interaction between lifestyle, physiological factors, and the gut microbiome. However, there is a lack of comprehensive reviews synthesizing this information specifically in the context of cancer patients. We need to map the existing literature to understand this complex interaction, identify knowledge gaps and inform future research directions aimed at improving gut microbiome-targeted interventions for cancer patients. This review and network analysis aims to address the gap via two main aims: (1) To explore the effects of physical activity and diet on the gut microbiome among cancer patients, and (2) To examine how non-lifestyle factors such as demographic, physiological and clinical factors may influence the gut microbiome among cancer patients.

## RESULTS

### Study selection

**Figure 1:**
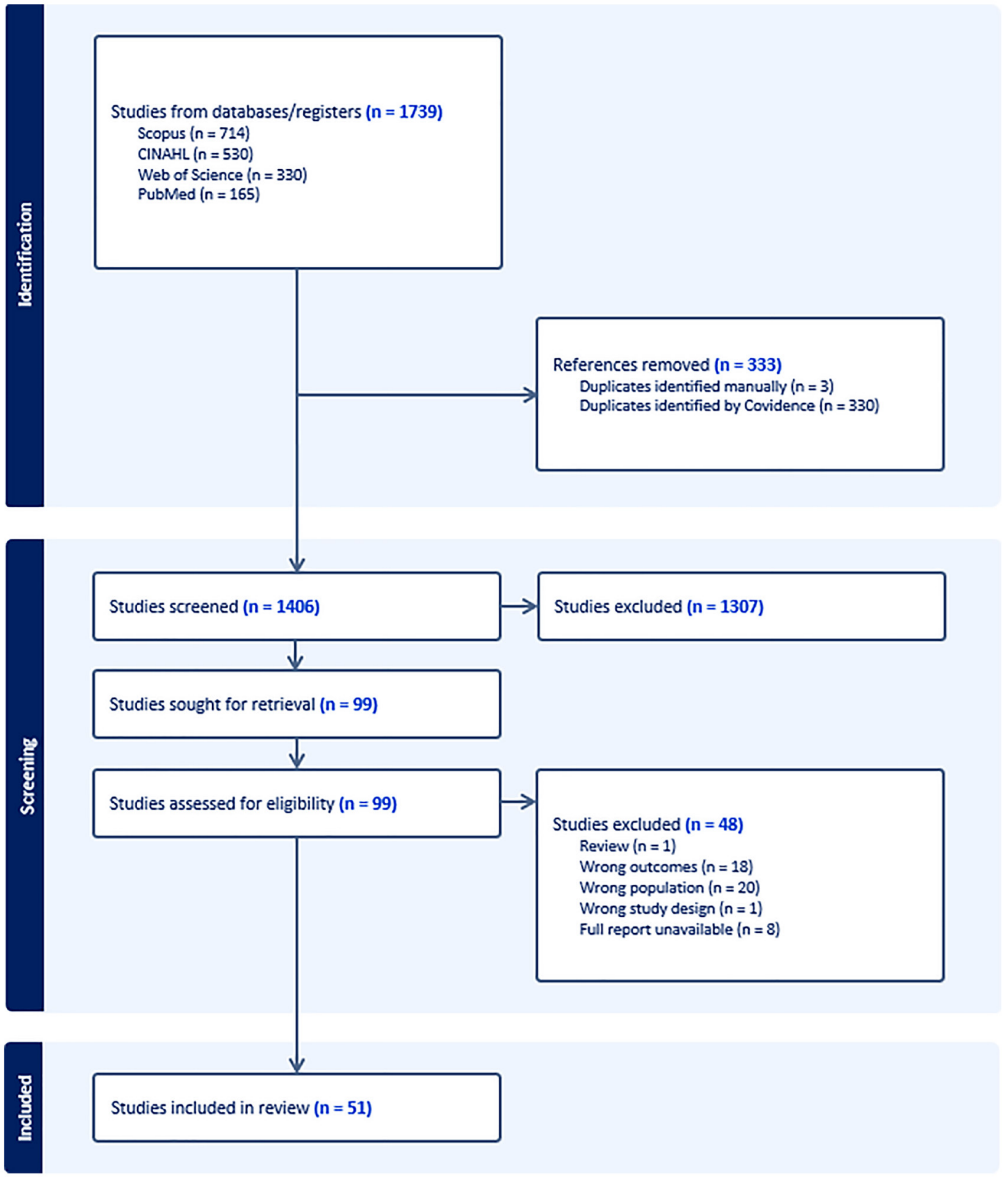
PRISMA diagram of process.

The database search yielded a total of 1739 studies, where 333 duplicates were removed. Primary screening of the remaining 1406 studies which involved reading their titles and abstracts to determine eligibility resulted in 99 studies for full-text screening. The full-text screening excluded 48 studies with reasons of exclusion such as wrong outcomes, wrong population, wrong study design, and full report unavailable. Findings from the remaining 51 studies were synthesized for this review and network analysis. A summary of the studies has been presented in Supplementary Table 1. Interrater reliability was assessed using Cohen’s kappa (κ), yielding κ = 0.58 for the title/abstract screening stage (moderate agreement) and κ = 0.94 for the full-text screening stage (almost perfect agreement), indicating improved reviewer concordance after initial calibration. A PRISMA diagram was used to present the flow chart of the process (Figure 1).

### Characteristics of studies

**Figure 2:**
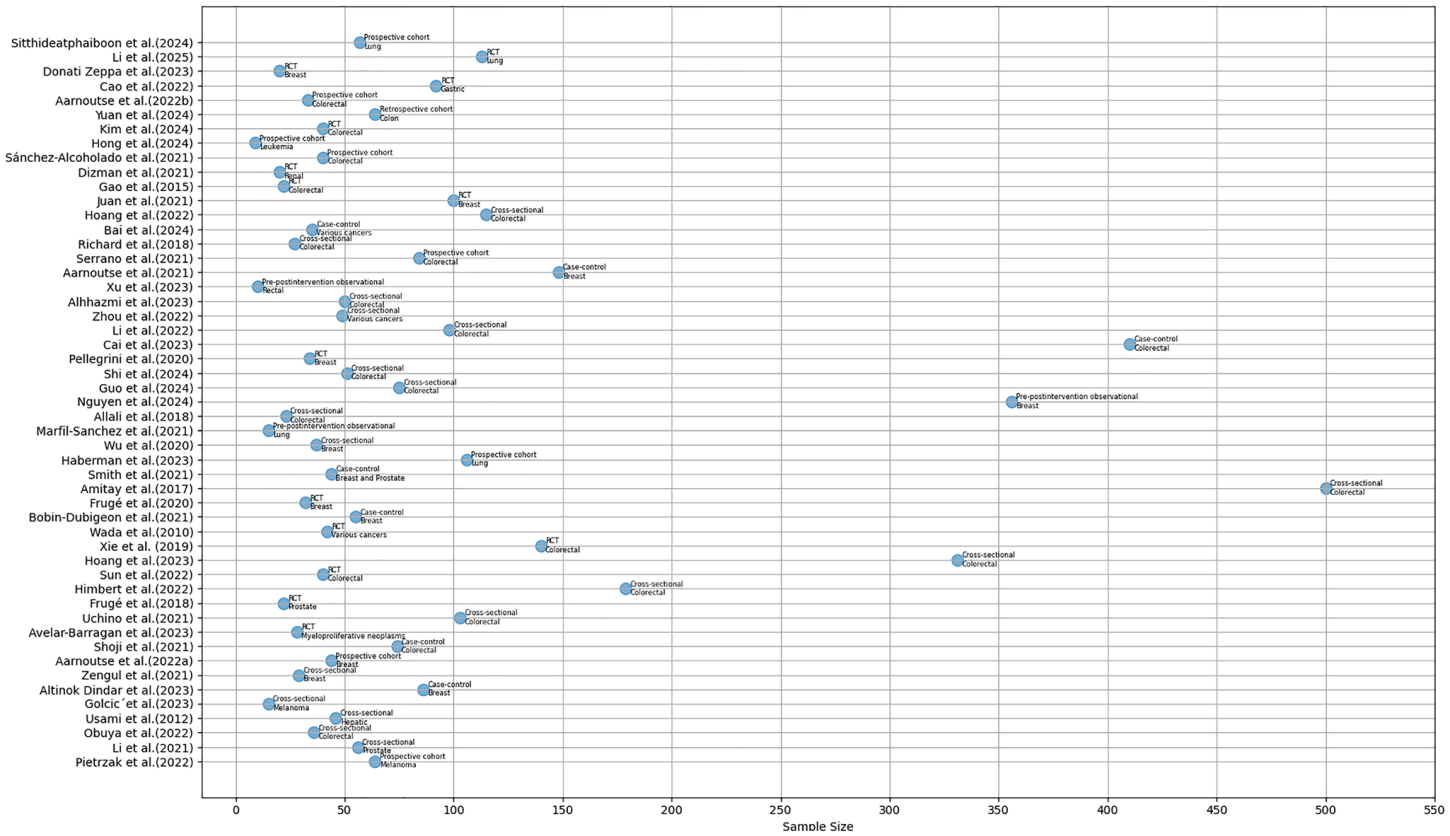
Summary of study distribution and characteristics.

Most of the studies had a sample size of less than 100, and only four studies had over 300 participants [64–67]. The top three designs used among the studies were cross-sectional (*n* = 18), randomized controlled trials (*n* = 14) and prospective cohort (*n* = 8) designs. Almost all the studies (*n* = 48) used 16s rRNA and/or shotgun metagenomic sequencing approaches, and only 3 studies [68–70] used culturing and polymerase chain reaction (PCR). Most studies (*n* = 21) involved participants with colorectal cancer, followed by breast cancer (*n* = 11). The summary of study characteristics has been presented in Figure 2.

### Associations between physical activity and the gut microbiome

Three studies reported that physically active cancer patients have a distinct microbial composition compared to inactive cancer patients [71–73]. Patients with higher PA levels, characterized by higher International Physical Activity Questionnaire (IPAQ) scores or having activity levels greater than 8.75 metabolic equivalent task (METs) hours per week, generally had higher alpha diversities compared to low activity or inactive groups (*p* < 0.05) [71–73]. Higher PA levels was positively correlated to increased abundance of SCFA producers such as *Phascolarctobacterium* and *Ruminococcaceae* (*p* < 0.05) [72]. These bacteria are known to generate propionate and butyrate, metabolites that support epithelial barrier integrity, exert antiinflammatory effects, and modulate immune cell differentiation. Higher PA was also associated with potential pathogens such as *Megasphaera*, although this correlation was not statistically significant [72]. Moreover, active patients had higher abundances of saccharolytics such as *Faecalibacterium* and *Blautia* [71], both key butyrate producers involved in maintaining mucosal homeostasis and downregulating pro-inflammatory signaling pathways. In contrast, inactive patients had lower abundances of saccharolytic *Lachnobacterium* and lactic-acid producing bacteria *Lactobacillus.* Interestingly, the inactive group had higher levels of *Veillonella* (*p* < 0.005) [73], a lactate-utilizing genus that can convert lactate to propionate although its role in cancer populations remains unclear.

Overall, these studies provide preliminary evidence that PA may shift the gut microbiome toward a more metabolically favorable and anti-inflammatory profile. However, two of these studies were descriptive, comparing active versus inactive groups without examining dose-response relationships [71, 73], and only one study evaluated direct correlations between PA levels and specific microbial taxa [72].

### Associations between diet and the gut microbiome

#### Plant-based diet

Plant-based diets may promote a gut microbiome profile that supports anti-inflammatory processes and gut health. Higher intake of flavones and anthocyanin, which are plant-derived compounds, is positively correlated with increased abundance of *Lactobacillaceae* (r = 0.54, *p* = 0.04) and *Ruminococcaceae* (r = 0.56, *p* = 0.03) [74]. Also, cross-sectional findings from Hoang et al. [75] found that seaweed intake was associated with 6% and 7% lower abundances of *Rikenellaceae* (95% CI, 2% to 11%) and *Alistipes* (95% CI, 2% to 11%) respectively. These gut microbial changes indicate transition to a healthier state because *Lactobacillaceae* and *Ruminococcaceae* are SCFA producers whose metabolites improve gut integrity whiles *Rikenellaceae* may be pathogenic. These changes may also improve response to chemotherapy because a prospective study by Pietrzak et al. [76] found that responders who had a higher intake of plant-based foods exhibited higher levels of other saccharolytic and SCFA producers such as *Prevotella* and *Bacteroides*.

#### Mediterranean diet

Evidence indicates that adherence to a mediterranean (MD) diet, which focuses mainly on whole, plant-based foods and healthy fats confers benefits to cancer survivors. A randomized controlled trial demonstrated that MD diet may improve gut homeostasis and immune function, as reflected by an enrichment of beneficial bacteria such as *Lachnospiraceae, Faecalibacterium*, and *Butyricimonas* [77]. The benefits of MD diet are further amplified when combined with aerobic exercise: the dual intervention reduced the abundance of pathogenic *Proteobacteria* (effect size (d) = −0.701, *p* = 0.006) while increasing the abundance of lactic-acid producing *Lactobacillales* (d = 0.459, *p* = 0.04), which suggests reduced inflammation and improved gut health [77].

#### Dairy

In a case-control study of breast cancer patients, lower dairy intake, as assessed by the Healthy Eating Index (HEI-2015) dairy component score was associated with an increased abundance of *Hungatella* (*p* = 0.029), which could promote a pro-inflammatory state [78].

#### Vitamins

Intake of vitamins may promote a beneficial gut profile and aid in treatment efficacy; however, these findings are not consistent across studies. Vitamin D intake was positively correlated with an increased abundance of *Bifidobacterium pseudocatenulatum* in cancer patients [74]. Low levels of 25-hydroxyvitamin D (25-OHD) among a significant proportion of CRC patients was linked to a higher abundance of *Parvimonas* genus which are often associated with opportunistic infections [79]. On the other hand, pediatric cancer patients had a less balanced gut ecosystem because adequate vitamin B6 intake was associated with a higher Chao1 diversity index, but vitamin A intake had a negative trend with Pielou’s evenness index [80].

#### Fruits and vegetables

A case-control study found that lower whole fruit consumption and higher vegetable intake was associated with an increased presence of *Acidaminococcus* (*p* = 0.005) and *Hungatella* (*p* = 0.024) respectively [78]. *Acidaminococcus* is associated with protein fermentation and generally considered to be a commensal in the gut, unlike *Hungatella* which may be pathogenic. This may imply that intake of fruits and vegetables may reduce abundance of pathogenic bacteria, however, further evidence is required to support these preliminary findings.

#### Fiber

Evidence suggests that dietary fiber may support the integrity of the gut barrier and support metabolic and gut health. Higher intake was inversely associated with *Clostridium* but positively correlated with *Bacteroides, Bifidobacterium* and *Prevotella* species which support SCFA production and immune health [65, 81]. In addition, an RCT found that increased dietary fiber consumption was positively correlated with a higher abundance of the mucin producing *Akkermansia* (r = 0.626, *p* = 0.002) [82], and in a cross-sectional finding among pediatric cancer patients, high fiber group had a greater presence of the commensal *Cyanobacteria* [80].

#### Red meat and poultry

An RCT from Frugé et al. [83] reported an association between higher red meat intake and reduction in the abundance of the saccharolytic, *Prevotella* (rho = 0.497, *p* = 0.018) and an increase in *Blautia* abundance (rho = 0.422, *p* = 0.039). They also found that increased poultry intake was associated with a reduction in the abundance of the saccharolytic, *Clostridiales* (*p* = 0.009) [83].

#### Prebiotic supplement

Xie et al. [84] conducted an RCT to investigate the effects of prebiotics on intestinal microbiota structure in perioperative CRC patients. They found that prebiotic supplementation group had a higher abundance of the SCFA producer, *Bifidobacterium* (*p* = 0.017), and pathogenic *Enterococcus* (*p* = 0.02), while reducing pro-inflammatory *Bacteroides* levels (*p* = 0.04).

#### Probiotic supplement

Probiotic interventions are associated with increased microbial diversity, as evidenced by higher Chao and Abundance -based coverage estimator (ACE) indices, and an elevated number of bacterial species in intervention groups [85, 86]. Specific microbial shifts included increased abundances of beneficial species of *Clostridium*, *Proteobacteria*, *Tenericutes*, *Eubacterium*, *Bifidobacterium*, *Lactobacillus, Barnesiella*, *Bacteroides, Faecalibacterium, Gemmiger* and *Akkermansia*, particularly in patients achieving clinical benefits like partial response or stable disease [69, 85, 87–90]. Conversely, probiotic supplementation was associated with reduced abundances of pathogenic species of *Enterococcus*, *Bacteroides*, *Streptococcus, Desulfovibrio, Actinomyces*, *Anaerostipes*, *Escherichia* and *Fusobacterium* [69, 85, 87, 89].

#### Fats

Higher polyunsaturated fatty acids (PUFAs) may potentially promote a beneficial gut through its positive correlation with increased abundance of *Akkermansia* (rho = 0.512, *p* = 0.003) [82]. In pediatric cancer patients, inadequate fat intake may potentially lead to microbial imbalances such as increased pathogenic *Megasphaera* which could disrupt gut health, while adequate fat intake supports beneficial species of *Erysipelotrichaceae* and *Peptostreptococcaceae* with mixed effects [80]. Finally, consumption of animal fat and fatty acids has shown negative correlations with the relative abundance of *Bacteroides* (rho = −0.27) and *Clostridium* (rho = −0.30 to −0.24) [75].

#### Micronutrients

Zhou et al. [80] found that beta-carotene intake was positively correlated with Faith’s Phylogenetic Diversity (Faith’s_PD) (*p* = 0.02) and showed a trend toward positive association with the Chao1 diversity index (*p* = 0.08) in pediatric cancer patients, while selenium intake was negatively correlated with both Shannon’s diversity index (*p* = 0.05) and Pielou’s evenness index (*p* = 0.06).

### Sterile diet

A sterile diet, also called neutropenic diet, is a diet that is low in microbes and designed for individuals with compromised immune systems. Hong et al. [91] found this diet to significantly reduce microbial diversity with an 85.33% decrease in microbial interaction network edges. However, microbial richness and interactions were observed to recover following the transition to a normal diet.

### Modified microbiota-accessible carbohydrate diet

A modified microbiota-accessible carbohydrate (mMAC) diet influenced some aspects of the gut microbiome in the intervention group without broadly affecting microbial diversity. No significant changes in alpha- or beta diversity were observed between pre- and post-diet samples; however, the mMAC diet significantly increased the abundance of *Prevotella* (*p* = 0.0218), which is known in the production of short-chain fatty acids, specifically acetate and propionate [92].

### Associations between non-lifestyle factors and the gut microbiome

#### Inflammatory markers

Avelar-Barragan et al. [93] found significant correlations between increased levels of pro-inflammatory cytokines, such as TNFα, IL-12p70, and IL-8, and specific microbial taxa, with TNFα positively correlated with *Flavonfractor* (rho = 0.39, *p* = 0.038), IL-12p70 negatively with *Roseburia* (rho = −0.55, *p* = 0.002), and IL-8 with *Eubacterium*.

#### BMI and obesity

Studies reported associations between obesity, body mass index (BMI), and total body fat (TBF) with alterations in gut microbiome composition [67, 68, 71, 73, 94]. Obese patients are likely to have a less diverse, pro-inflammatory gut environment with increased abundance of pathogenic species of *Faecalibacterium*, *Blautia*, *Sutterella*, *Clostridiaceae, Clostridium, Lachnospira*, and *Verrucomicrobia*, but reduced abundance of beneficial species of *Enterococcus*, *Lactobacillus*, *Streptococcus*, *Actinobacteria*, *Clostridium, Succinivibrio*, and *Firmicutes* [67, 71, 73, 94]. Higher BMI and total body fat are also associated with reduced microbial diversity and more homogeneous microbial communities [67, 68, 73].

#### Chemotherapy

Studies have reported chemotherapy to be associated with decreased alpha diversity alongside a significant decline in species richness [95, 96]. Beneficial bacteria such as *Ruminococcaceae*, *Christensenellaceae*, *Marvinbryantia*, and *Bacillus* deplete during treatment, while *Proteobacteria*, *Enterobacterales*, *Firmicutes and Lactobacillus* increase, with some taxa recovering post-treatment [69, 95, 97]. Additionally, Bai et al. [96] revealed that *Megasphaera* and *Prevotella* were elevated in cancer patients pre- and post-chemotherapy, while negative associations were observed with *Lactobacillus*, *Bifidobacterium*, and *Roseburia*. One study also found that although capecitabine treatment showed no significant changes in major phyla, high baseline *Bifidobacterium* levels were associated with better tumor response [98].

### Cancer type and stage

#### Colorectal cancer

Colorectal cancer (CRC) patients consistently exhibit lower gut microbial diversity compared to healthy controls, as reported by multiple studies [64, 66, 79, 99–105]. A hallmark of CRC-related dysbiosis is the depletion of beneficial SCFA–producing bacteria such as *Faecalibacterium*, *Blautia*, *Roseburia*, *Agathobacter*, *Dorea*, and *Subdoligranulum*, particularly at tumor sites [99, 100, 106]. At the same time, multiple pathogenic or pro-tumorigenic taxa are enriched in CRC. *Fusobacterium* is one of the most consistently elevated genera [66, 79, 101, 102, 106], correlating with fecal occult blood test (FOBT) positivity and advanced disease [106], though it is has been reported to be depleted in colitis-associated cancer (CAC) patients [100]. Other pathogenic or potentially pro-tumorigenic bacteria enriched in CRC include *Escherichia-Shigella*, *Enterococcus*, *Proteobacteria*, *Pseudomonas*, *Peptostreptococcus*, *Streptococcus*, *Parvimonas*, *Solobacterium*, and *Bacteroides* [79, 99, 101–105]. Additionally, other pathogenic genera such as *Klebsiella* and *Enterobacter*, were significantly overrepresented in CRC patients, particularly in CAC [64, 100, 104]. Pathogenic *Streptococcus* species have been reported to be enriched in CAC, and this further supports their role in CRC development [100]. Tumor stage and location further shape microbial profiles. Advanced CRC stages (III and IV) show higher abundances of *Fusobacterium*, *Solobacterium*, *Rothia*, *Coprobacillus*, *Veillonella*, *Sellimonas* and *Bacteroidetes* taxa, including *Prevotella*, *Alistipes*, *Alloprevotella*, and *Odoribacter* [64, 66, 92, 103]. Stage-specific patterns include elevated *Rothia*, *Coprobacillus*, and *Veillonella*, and increased *Sellimonas* and *Eubacterium* in left-sided tumors [92]. In contrast, early-stage CRC (I and II) show less pronounced microbial shifts, with *Fusobacterium* not implicated in these earlier lesions [66]. Subtype analyses reveal that type I CRC and colorectal adenomas are characterized by enrichment of *Escherichia-Shigella*, *Bifidobacterium*, *Bacteroides*, *Flavonifractor*, *Tyzzerella*, and *Lachnoclostridium* [64]. There were other microbial patterns observed in the tumor mucosal microbiota which was enriched with CRC-promoting bacteria (*Fusobacterium*, *Gemella*, *Campylobacter*), while para-cancerous mucosa showed intermediate microbial characteristics [106].

#### Breast cancer

Breast cancer (BC) patients have reduced gut microbial diversity as evidenced by a significantly lower Shannon diversity index [73, 107], though one study found no significant differences in species richness or Shannon index [108]. They also exhibited gut microbial profiles that were pro-inflammatory and tumor-supportive, with higher abundances of specific *Firmicutes* taxa (*Clostridium* cluster IV, *Clostridium* cluster XIVa, *Blautia*) and *Allobaculum*, but lower *Bifidobacterium*, *Odoribacter, Butyricimonas*, and *Coprococcus* compared to controls [73, 107, 109]. In terms of the BC subtypes, there are inconsistent findings across studies such as no significant associations between microbial diversity and BC subtypes [65]. However, Wu et al. [73] reported that HER2+ patients had higher abundances of *Alistipes*, *Enterococcus*, and *Acidaminococcus*, while *Rikenellaceae*, *Methanobrevibacter*, *Christensenellaceae*, *Turicibacter*, *Clostridium*, *SMB53*, *Blautia*, *Coprococcus*, *Ruminococcus*, and *Desulfovibrio* were less abundant compared to HER2− patients [73]. For estrogen receptor (ER) status, *Enterococcus*, *Turicibacter*, *Veillonella*, and *Haemophilus* were less abundant in ER-positive patients. Similarly, progesterone receptor (PR)-positive patients had lower abundances of *Turicibacter*, *Clostridium* (from *Clostridiaceae* and *Erysipelotrichaceae*), compared to PR− patients [73]. Higher stage and grade BC has been associated with increased abundances of *Clostridium*, *Veillonella*, *Clostridiaceae*, *Eggerthella*, *Enterobacteriaceae*, and *Haemophilus*, but lower abundances of *Acidaminococcus, Coriobacteriaceae*, *Lachnospiraceae*, *Anaerostipes*, *Ruminococcaceae* and *Catenibacterium* [73]. Finally, increasing clinical tumor size and stage shown to be negatively correlated with *Veillonellaceae* and *Dialister* [108], implies a progressive decline in these taxa with disease progression.

#### Prostate cancer

A study found that prostate cancer (PCa) patients had higher alpha diversity and distinct beta diversity compared to cancer-free controls. The gut microbiota of these patients was enriched with *Tissierellaceae*, *Lachnospiraceae*, and *Ruminococcaceae* [109].

#### Lung cancer

Beneficial taxa, including *Clostridiales*, *Lachnospiraceae*, and *Faecalibacterium*, have been reported to be less abundant in LC patients, while *Akkermansia* correlated with better clinical outcomes (HR 4.26, 95% CI 1.98–9.16) and *Clostridium* with poor prognosis [110].

#### Surgery

Surgical interventions significantly alter the gut microbiome which affects microbial diversity, composition and interspecies relationships. Breast cancer surgery leads to reduction in gut microbial diversity as evidenced by postoperative stool samples which showed lower alpha and beta diversity compared to preoperative samples [65]. Lung resection surgery may have an influence on microbial shifts with increased abundance of *Alistipes* and *Bacteroides*, bacteria which are associated with anti-inflammatory processes through short-chain fatty acid production [111]. In addition to that are dynamic changes in species co-occurrence relationships with a shift from negative to positive correlations postoperatively for species of *Gemella*, *Adlercreutzia*, *Lachnospiraceae*, *Parabacteroides*, *Klebsiella*, and *Barnesiella* [111]. These dynamic changes in microbial interactions are indicative of the complex impact of surgical interventions on gut ecosystem stability. Evidence also suggests that radical colon cancer resection disrupts microbial balance which could compromise gut barrier function and immune homeostasis. This imbalance includes a decrease in beneficial genera such as *Bifidobacterium*, *Lactobacillus*, and *Enterococcus* and an increase in *Escherichia* and yeast populations [70].

#### Diagnosis delay

Nguyen et al. [65] reported that breast cancer experiencing diagnosis delays exhibited marked reduction in gut microbial diversity, alongside an elevated relative abundance of *Enorma*, and a concomitant decline in *Faecalicoccus*. The shift toward *Enorma* may reflect opportunistic expansion, while loss of *Faecalicoccus* could compromise beneficial functions such as SCFA synthesis, thereby potentially affecting host metabolic and immune homeostasis.

#### Menopause and menarche age

Menopausal stage shows no significant association with gut microbiome diversity [65]. However, early menarche (≤11 years) may increase risks of metabolic or inflammatory disorders due to reduced microbial diversity and lower Firmicutes abundance, with specific decreases in *Coriobacteriaceae, Methanobrevibacter*, and several *Firmicutes* genera including *Turicibacter, Anaerostipes, Ruminococcus*, but increased *Lachnospiracaceae, Clostridium* and *Escherichia* [73].

#### Hormone therapy

Androgen deprivation therapy (ADT) may promote systemic inflammation and gut dysbiosis which could exacerbate metabolic disorders because it is associated with increased levels of pro-inflammatory bacteria including pathogenic species of *Ruminococcus* and *Bacteroides*, and decrease in beneficial genera including *Lachnospira* and *Roseburia* [112].

#### Age

Younger patients’ microbiomes may support diverse metabolic functions due to enriched taxa like *Lactobacillus* (probiotic) and *Prevotella* species involved in fiber metabolism. On the other hand, older patients’ increased pathogenic species of *Clostridium* and *Bacteroides* may reflect shifts toward pro-inflammatory or pathogenic profiles which increases risks of gut-related diseases [101].

#### Radiotherapy and radiochemotherapy

Evidence show that neoadjuvant radiotherapy (NART) does not significantly affect alpha diversity but alters bacterial composition. Prolonged NART reduces cancer-associated genera (*Enterobacter, Citrobacter, Peptoniphilus, Dialister, Intestinibacter*) and increases oral pathogenic bacteria (*Streptococcus, Shuttleworthia, Parascardovia, Lachnoanaerobaculum*) [113]. Additionally, responders to radio chemotherapy show higher microbial diversity than non-responders post-treatment, with increased beneficial bacteria (*Bifidobacterium, Ruminococcus, Roseburia, Faecalibacterium*) and reduced proinflammatory/pathogenic species of *Fusobacterium, Bacteroides, Escherichia, Prevotella, Klebsiella* [114].

### Network analysis

**Figure 3:**
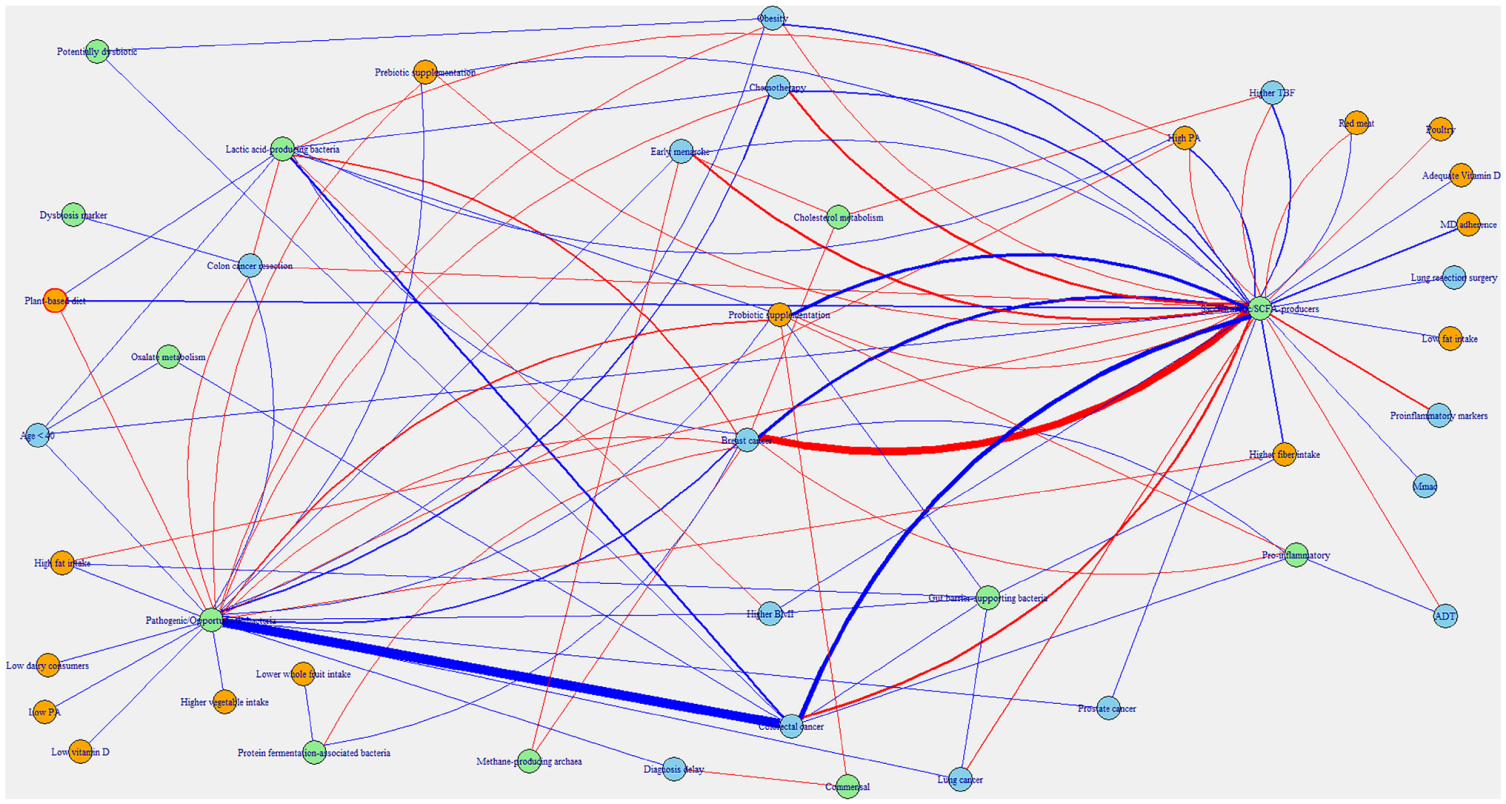
Network diagram of associations between diet, physical activity and non-lifestyle factors. Orange node: Diet and PA; Blue node: Non-lifestyle factors; Green node: Cluster of bacteria function. Orange and blue nodes are connected to green nodes via edges. Blue edge: Increase; Red edge: Decrease.

The chi-squared test of independence showed that the distribution of bacteria function was associated with the factors examined (Χ^2^ = 390.87, *p* = 0.032). The network diagram in Figure 3 reveals that the bacteria mainly reported in the literature are saccharolytic/SCFA producers and pathogenic/opportunistic bacteria. The diagram indicates that there are inconsistent findings across studies, for instance, breast and colorectal cancers both increase and decrease saccharolytic/SCFA producers and pathogenic/opportunistic bacteria. However, a focus on the edges shows that breast cancer is more associated with a reduction in saccharolytic/SCFA producers and colorectal cancer increases pathogenic/opportunistic bacteria. The network shows that the effects of physical activity and diet on the gut microbiome are not independent of clinical, demographic and physiological characteristics of participants.

## DISCUSSION

The scoping review and network analysis aimed to explore the associations between physical activity, diet and the gut microbiome of cancer patients, with an examination of clinical, demographic and physiological factors that may influence these associations. Higher PA levels was associated with increased gut microbial diversity, and higher abundances of beneficial bacterial taxa. Healthy diets such as plant-based, mediterranean, dietary fiber, probiotics, prebiotics, fruits and vegetables were consistently associated with increased abundances of beneficial bacteria such as SCFA-producers which have anti-inflammatory properties and improve gut integrity. Moreover, clinical, demographic and physiological characteristics such as chemotherapy, age, BMI, diagnosis delay, menopause, surgery, hormone and radiation therapy were associated with extensive gut microbial fluctuations.

Many studies have explored the effects of physical activity and dietary modifications as lifestyle interventions in mitigating cancer symptoms and improve outcomes. There is extensive literature supporting the evidence that these interventions are effective in improving cancer symptoms [115–118]. Despite the extensive literature, interactions between physical activity and the gut microbiome specifically in cancer population remain largely underexplored. This review found only 3 studies which reported that high physical activity is associated with increased gut microbiome diversity, decreased abundances of pathogenic bacteria and increased abundances of bacteria associated with SCFA production. Although some evidence exists to support these associations, a clarification on the dose-response relationships (intensity, frequency, duration) in future studies may be useful in modulating microbiome-driven symptom burden [119]. Dietary modifications provide nutritional requirements needed to supplement the benefits of high physical activity [120]. However, the ideal diet for all cancer patients does not exist because most of these dietary plans need to be personalized for maximum gains. According to this review, plant-based diets, probiotic supplementation and adherence to a mediterranean diet are gut supportive and could improve health outcomes of cancer patients. Probiotics, for instance, typically contain SCFA and/or lactic acid producing or associated bacteria [121], hence increasing production of these metabolites in the gut while decreasing pathogenic bacteria. Therefore, dietary modulation of the gut microbiome, particularly through SCFA-supporting and lactic-acid supporting foods, is a viable strategy for symptom control and inflammation reduction in cancer patients. On the other hand, dietary components associated with pathogenic/opportunistic bacteria such as red meat, high fat intake, low fiber intake and low vitamin D shift the microbiome toward proteolytic fermentation which produces harmful metabolites such as ammonia and hydrogen sulfide that drive inflammatory responses and impair gut mucosal health [122–124].

The beneficial effects of physical activity and dietary modifications are affected by several nonlifestyle participant characteristics which have independent effects on the gut microbiome. These non-lifestyle characteristics could drive gut dysbiosis which masks the benefits of lifestyle interventions [125]. For example, the network diagram showed that chemotherapy and ADT have been associated with reductions in microbial diversity and depletion of SCFA-producers, while increasing pro-inflammatory and opportunistic taxa. Also, age-related changes in gut physiology and immune function can shift microbial communities in ways that influence responsiveness to lifestyle-based interventions [126, 127]. Their effects on the gut microbiome may contribute to the wide interindividual variability in gut microbiome profiles observed among cancer patients.

This variability complicates interpretation of intervention outcomes and highlights the need for personalized or stratified approaches to some extent in microbiome research. Most of these factors could act as cofounders and accounting for all of them in future study designs using traditional statistical approaches may not be entirely feasible because of high dimensionality, multicollinearity, and complex interdependencies among variables [128, 129]. Moreover, many of these cofounders are not easily controlled or randomized and stratifying for all of them would require large sample sizes that may not be practical in most clinical and interventional trials. These challenges highlight the need for advanced modeling techniques such as machine learning and causal inference frameworks that can handle multi-variable interactions and latent confounding [130, 131]. Designing a sufficiently powered study that would have such detailed metadata would require a huge sample size, substantial financial resources and extensive logistical coordination. The NIH Nutrition for Precision Health program, powered by the All of Us research program, may provide opportunities in the future to design such comprehensive, multi-dimensional studies [132]. This could enable more accurate characterization of dose-response relationships, inter-individual variability, and mechanistic pathways linking diet, physical activity, and the gut microbiome to cancer outcomes and symptom management.

The network analysis aimed to map associations reported in the literature; however, it treated all studies as equally weighted, despite differences in sample sizes and methodological quality. This may have introduced bias by giving equal influence on studies of varying rigor and statistical power. Moreover, several factors (e.g., different BC subtypes) were clustered into single factors to improve network visibility and interpretability. However, this simplification led to a loss of granularity and obscured subtype-specific associations. Most studies had small sample sizes of less than 100, with a significant number of the RCTs and prospective cohort studies falling below 50. A primary reason for this is that the incorporation of the gut microbiome into studies is very expensive and may not always be feasible for large scale studies. These costs are not limited to sample collection and sequencing alone but extend to costs incurred in bioinformatics and data processing [133, 134]. Whole metagenomic sequencing and longitudinal microbiome profiling demand considerable financial and computational resources, often making such studies impractical at scale. This limitation is further compounded by participant burden, inconvenience and lack of adherence to provision of stool samples which makes it more challenging, especially for RCTs and prospective cohort designs [135–137]. Finally, this study’s focus on the gut microbiome omits the impact of oral microbiome. Oral microbiome translocation to the gut is a phenomenon that can modulate the gut microbiome and may be involved in various health outcomes among cancer patients [138]. Future studies on this oral-gut microbiome axis may provide further insights into its role in cancer progression, treatment response and microbial diversity.

## MATERIALS AND METHODS

### Search strategy

A search was conducted on Scopus, CINAHL, PubMed, and Web of Science databases using keywords guided by the PICO framework [139], on October 21, 2024, and May 16, 2025. The second search was a continuation of the first one to ensure any published literature within that period was captured. The search algorithm was developed using MeSH terminologies and keywords and their synonyms from related articles (refer to Appendix A).

### Screening and study selection

Output from the databases were imported into Covidence (a web-based platform that streamlines the conduct of literature reviews) for screening and selection. Eligibility of studies was defined using the criteria detailed in Table 1. The first step involved title and abstract reading to determine eligible studies. The identified studies were moved to the next step, which was full text reading to generate a final list of studies for the review. These processes were conducted by two independent reviewers (J.A and S.A) and disagreements were addressed through meetings and discussions until consensus was reached. An interrater reliability was calculated for both steps (abstract and full-text screening) with Cohen’s kappa coefficient.

**Table 1:** Eligibility criteria for screening studies

	Inclusion	Exclusion
Population	All cancer patients, including both children and adults.	Animal models and non-cancer patients.
Intervention	Studies involving the measurement or monitoring of diet and/or physical activity levels.	
Comparator	None.	
Outcome	Studies that report changes in gut microbiome.	Studies focusing on only patient reported outcomes and not including the gut microbiome.
Study characteristics	All observational studies.	Non-peer reviewed articles, case reports, other reviews and editorials.
Other	Studies considering clinical, demographic or physiological factors that have effects on gut microbiome such as chemotherapy, radiotherapy, estrogen levels, surgery, comorbidities, sleep, age, gender, ethnicity, and fatigue.	

### Data extraction and analysis

Data extracted from included studies comprised country, participants, study design, cancer type, microbiome analysis, and main findings. Findings from the studies were summarized under three main categories: (a) Physical activity and the gut microbiome. (b) Diet and the gut microbiome. (c) Clinical, demographic and physiological factors influencing the gut microbiome.

For quantitative synthesis, the extracted data was pooled for further analysis by categorizing them into factors, microbiome outcome, effect and bacteria function. Factors referred to physical activity, diet, and non-modifiable participant features that were reported by the studies. Microbiome outcomes were the bacterial taxa whose changes (effect: increase or decrease) were associated with the factors. Bacteria function referred to the processes in which these bacteria were involved in such as SCFA production, lactic acid production, pathogenic/opportunistic.etc. To evaluate whether bacterial function was associated with factor type, we constructed a contingency table with bacterial function categories as rows and factor categories as columns, counting each reported association as one observation. A chi-squared test of independence was conducted to check if the distribution of bacteria function was dependent on the factors. A network diagram was used to map the associations between factors and bacterial function to explore the direction of their effects (Figure 2). Each study was assigned the same weight, hence nodes of equal sizes but unique color codes were used to represent bacterial function, lifestyle and non-lifestyle factors. Also, the amount of evidence connecting factors to bacterial functions determined the thickness of edges, with thicker edges representing commonly reported associations by studies.

## CONCLUSIONS

This review and network analysis highlights the emerging but still limited evidence linking physical activity, diet, and the gut microbiome in cancer populations. While lifestyle interventions such as physical activity and dietary modulation show promise in improving gut microbial profiles and improving cancer outcomes, current research is constrained by small sample sizes, high costs, and methodological challenges. Inter-individual variability driven by non-lifestyle factors such as treatment regimens and physiological differences further complicates interpretation and generalization of findings. Currently, gut microbiome tests are of limited value to support robust microbiome-supported clinical education and practice [140]. In parallel, non-invasive technologies like stool-based DNA screening tools (e.g., Cologuard) and other microbial or metabolite-based diagnostics such as fecal immunochemical tests (FIT), circulating tumor DNA (ctDNA) assays, metagenomic sequencing-based early detection platforms, and multi-omics screening panels illustrate the potential for future clinical applications. Even though these technologies demonstrate significant progress in early cancer detection and risk stratification, they remain limited in their ability to characterize lifestyle-driven microbial changes or provide mechanistic insight into how physical activity or diet shape cancer-related microbiome profiles. To advance this field, future studies must adopt more comprehensive and scalable designs, integrate advanced analytical methods, account for complex confounders, and clarify the roles and functions of specific gut bacteria and associated metabolites. Future directions should also focus on developing standardized protocols for lifestyle-microbiome intervention studies, enhancing longitudinal monitoring through multiomics, and creating cost-effective, scalable microbiome profiling approaches that can be integrated with lifestyle data. Finally, harmonized data-sharing frameworks, and large diverse cohorts will be essential to reduce current research gaps and improve translation into clinical practice.

## SUPPLEMENTARY MATERIALS




