# Supplementary material for: Associations among physical activity, diet, non-lifestyle characteristics and the gut microbiome of cancer patients: A scoping review and network analysis
Source: Oncoscience. 2026 Mar 11;13:85–103. doi: 10.18632/oncoscience.651 (PMC12998689; doi:10.18632/oncoscience.651)
Supplement: Supplementary file 1 [file oncoscience-13-651-s001.pdf]

Associations among physical activity, diet, non-lifestyle characteristics and the gut microbiome of cancer patients: A scoping review and network analysis

SUPPLEMENTARY MATERIALS

Supplementary Table 1: Summary of studies. See Supplementary Table 1

APPENDIX

PubMed Search

Population - Cancer patients

Neoplasms [MeSH] OR Cancer Survivors [MeSH] OR Patients [MeSH] “cancer patients” OR “oncology patients” OR “individuals with cancer” OR “cancer survivors” OR “cancer sufferers” OR “breast cancer patients” OR “colorectal cancer patients” OR “lung cancer patients” OR “prostate cancer patients” OR “ovarian cancer patients” OR “leukemia patients” OR “lymphoma patients” OR “melanoma patients”

Intervention - Physical activity and diet

“Exercise”[Mesh] OR “Physical exercise” OR “Physical activity” OR “Aerobic exercise*” OR “Resistance Exercise*” OR “Isometric exercise*” OR “Acute exercise” OR “Acute training” OR “Exercise training*” OR Workout OR “Diet”[Mesh] OR “Carbohydrate loading” OR “Carbohydrate-restricted” OR “High-protein low-carbohydrate” OR “Dietary patterns” OR “Dietary modifications” OR “Dietary changes” OR “Vegetarian” OR “Plant-based” OR “Mediterranean diet” OR “Protein-restricted” OR “Caloric restriction” OR “Fasting” OR “Nutrition” OR “Nutritional therap*” OR “Nutritional change*” OR “Enteral nutrition” OR “Nutritional status”

Outcome - Gut microbiome

“Gastrointestinal Microbiome”[Mesh] OR ”Gut microbiome” OR “Gastrointestinal microflora” OR “Gut microflora” OR “Gastrointestinal microbiota” OR “Gut microbiota” OR “Gastrointestinal flora” OR “Gut flora” OR “Gastrointestinal microbial communit*” OR “Intestinal microbiome” OR “Intestinal microbiota” OR “Intestinal microflora” OR “Enteric Bacteria” OR “Gastric microflora” OR “Gastric microbiome”

Scopus, Web of science and CINAHL

Population - Cancer patients

Neoplasms OR “Cancer Survivors” OR “cancer patients” OR “oncology patients” OR “individuals with cancer” OR “cancer sufferers” OR “breast cancer patients” OR “colorectal cancer patients” OR “lung cancer patients” OR “prostate cancer patients” OR “ovarian cancer patients” OR “leukemia patients” OR “lymphoma patients” OR “melanoma patients”

Intervention - Physical activity and diet

“Exercise” OR “Physical exercise” OR “Physical activity” OR “Aerobic exercise*” OR “Resistance Exercise*” OR “Isometric exercise*” OR “Acute exercise” OR “Acute training” OR “Exercise training*” OR Workout OR “Diet” OR “Carbohydrate loading” OR “Carbohydrate-restricted” OR “High-protein low-carbohydrate” OR “Dietary patterns” OR “Dietary modifications” OR “Dietary changes” OR “Vegetarian” OR “Plant-based” OR “Mediterranean diet” OR “Protein-

restricted” OR “Caloric restriction” OR “Fasting” OR “Nutrition” OR “Nutritional therap*” OR “Nutritional change*” OR “Enteral nutrition” OR “Nutritional status”

Outcome - Gut microbiome

“Gastrointestinal Microbiome”[Mesh] OR ”Gut microbiome” OR “Gastrointestinal microflora” OR “Gut microflora” OR “Gastrointestinal microbiota” OR “Gut microbiota” OR “Gastrointestinal flora” OR “Gut flora” OR “Gastrointestinal microbial communit*” OR “Intestinal microbiome” OR “Intestinal microbiota” OR “Intestinal microflora” OR “Enteric Bacteria” OR “Gastric microflora” OR “Gastric microbiome”
