# Supplementary material for: Associations among physical activity, diet, non-lifestyle characteristics and the gut microbiome of cancer patients: A scoping review and network analysis
Source: Oncoscience. 2026 Mar 11;13:85–103. doi: 10.18632/oncoscience.651 (PMC12998689; doi:10.18632/oncoscience.651)
Supplement: Supplementary file 2 [file oncoscience-13-651-s002.docx]

**Supplementary Table 1: Summary of studies**

| **Author (Year)/Country** | **Objective** | **Participants** | **Main Findings** |
| --- | --- | --- | --- |
| Himbert et al. (2022)/USA and Germany | To investigate associations between physical activity, BMI, and gut microbiome diversity in CRC patients, and to assess whether physical activity may counteract gut microbiome dysbiosis due to obesity. | 179 patients with stage I-IV CRC, with pre-surgery stool samples analyzed. | Lower gut microbiome diversity was observed in inactive (<8.75 METs hrs/wk) vs. active (>8.75 METs hrs/wk) patients and obese vs. normal weight patients. Twelve genera and two phyla (Actinobacteria and Fusobacteria) showed significant differences across physical activity and BMI groups. *Faecalibacterium* and *Blautia* were higher in active patients, while *Clostridium* and *Succinivibrio* were lower in overweight/obese patients. *Actinobacteria* significantly increased in inactive and overweight/obese patients. |
| Sun et al. (2022)/China | To evaluate the association between PA, gut microbiome composition, and survival outcomes in metastatic CRC patients. | 40 stage IV colorectal cancer patients with unresectable metastatic CRC. | Patients with higher PA levels (high IPAQ scores) had a higher alpha diversity compared to middle PA group (p = 0.04). Higher PA levels were positively correlated to increased abundance of *Phascolarctobacterium* and *Ruminococcaceae* (p < 0.05) and negatively correlated with *Megasphaera* abundance but not significant (p > 0.05). |
| Wu et al. (2020)/ USA | To investigate associations between the gut microbiome, breast cancer risk factors (such as age at menarche, BMI, and total body fat), and breast tumor characteristics (including receptor status, stage, and grade). | 37 women newly diagnosed with invasive breast cancer from Los Angeles County, with fecal samples collected before chemotherapy. | HER2+ breast cancer patients had lower microbial diversity than HER2− patients, with higher Bacteroidetes (g_Alistipes) and Firmicutes (g_Enterococcus, g_Acidaminococcus) but lower abundances of other Bacteroidetes, Firmicutes, Euryarchaeota, and Proteobacteria; Firmicutes and Proteobacteria varied by ER/PR status, with higher-grade/stage cancers showing increased Firmicutes (g_Clostridium, g_Veillonella) and Proteobacteria but lower Actinobacteria and Firmicutes; early menarche (≤11 years), higher TBF (≥46%), and BMI (≥25 kg/m²) were linked to lower diversity, increased Firmicutes (f_Clostridiaceae) and Verrucomicrobia, and decreased Firmicutes (g_Lactobacillus, g_Streptococcus); physical activity group had higher Firmicutes (f_Clostridiaceae, g_Lachnobacterium, g_Lactobacillus) but lower Veillonella. |
| Pietrzak et al. (2022)/ Poland | To investigate the association between the response to anti-PD-1 therapy and the baseline gut microbiome in a cohort of melanoma patients. | The study included 64 melanoma patients who were enrolled for anti-PD-1 therapy and 10 healthy subjects who served as a control group. | Responders had a higher Bacteroidota to Firmicutes ratio and lower microbial richness. Responders' microbiota was enriched with Prevotella copri and Bacteroides uniformis. Higher consumption of plant-based foods was significantly associated with a positive response to therapy. |
| Li et al. (2021)/China | To investigate whether the gut microbiota composition differs between prostate cancer patients who had undergone androgen deprivation therapy (ADT) and those who had radical prostatectomy (RRP) | 56 patients who were undergoing ADT. 30 patients who had undergone radical prostatectomy (RRP). | Patients in the ADT group had significantly lower alpha-diversity compared to those in the RRP group. ADT patients had increased levels of *Ruminococcus gnavus* and *Bacteroides spp.*, which are associated with pro-inflammatory effects. Conversely, beneficial bacteria such as *Lachnospira* and *Roseburia* were reduced in the ADT group. |
| Obuya et al. (2022)/Kenya | To profile the gut microbiome of Kenyan colorectal cancer (CRC) patients and compare it with healthy individuals. | 18 CRC patients and 18 healthy controls. CRC patients were chemotherapy-naïve and had pathologically confirmed CRC. | CRC patients have reduced Prevotella copri and Faecalibacterium prausnitzii, with increased Bacillales, Tissierellaceae, Staphylcoccaceae, Helicobacteraceae, Mycoplasmataceae, and genera like Fusobacterium, Porphyromonas, Staphylococcus, and Veillonella. Enriched species include Bacteroides fragilis and others. Age differences: under 40 show more Xanthomonadales, P. copri, and Lactobacillus salivarius; over 40 have more Clostridium ramosum, Clostridium symbiosum, and Bacteroides ovatus. |
| Usami et al. (2012)/Japan | To analyze the relationships among intestinal microbiota, fecal organic acids, and plasma lipid composition in hepatic cancer patients with or without liver cirrhosis. | 46 hepatic cancer patients undergoing liver resection. Patients were classified into three groups based on histological findings of non-cancerous liver tissue: Normal Liver (NL): 14 patients. Chronic Hepatitis or Liver Fibrosis (CH/LF): 20 patients. | In the CH/LF group, Bifidobacterium, Enterobacteriaceae, Bacteroidaceae, Lactobacillus, and Enterococcus negatively correlated with serum free fatty acid, while Bifidobacterium, Bacteroidaceae, and Enterobacteriaceae also negatively correlated with serum formic acid; Candida positively correlated with serum phospholipids, Enterobacteriaceae negatively correlated with AA, Enterococcus positively correlated with fecal propionic acid, and BMI positively correlated with fecal Lecithinase-positive Clostridium. |
| Golcic´et al. (2023)/Croatia | To analyze the gut microbiome and dietary habits of metastatic melanoma patients who achieved a complete and sustained response to immunotherapy. | 15 metastatic melanoma patients who had been treated with immune checkpoint inhibitors (ICIs) and had a sustained complete response for at least 12 months. | Late responders consumed more flavones. Higher intake of anthocyanins, polyunsaturated fatty acids (PUFA), and vitamin D in late responders. Flavone intake positively correlated with Lactobacillaceae and Ruminococcaceae. Vitamin D intake correlated with Bifidobacterium pseudocatenulatum. Anthocyanin intake correlated with Firmicutes abundance. |
| Altinok Dindar et al. (2023)/USA | To investigate the association between gut microbiota composition and breast cancer (BC) while exploring diet as a potential modulating factor. | 42 newly diagnosed, treatment-naïve female breast cancer patients, and 44 age-matched cancer-free controls | Alpha diversity was significantly lower in BC patients compared to controls. Lower whole fruit intake was associated with higher *Acidaminococcus* presence. Lower dairy intake and higher vegetable intake were associated with higher *Hungatella* presence. |
| Zengul et al. (2021)/ USA | To examine associations between dietary fiber intake, gut microbiota composition, and estrogen metabolism in postmenopausal women with newly diagnosed BC (stage 0-II). | 29 postmenopausal women diagnosed with stage 0–II BC. | Higher dietary fiber intake, particularly soluble fiber, was inversely linked to Clostridium hathewayi and Clostridium, while insoluble fiber was positively associated with Bacteroides uniformis; in BC patients, Firmicutes and Bacteroidetes were the most abundant phyla, with Faecalibacterium more abundant and Bifidobacterium and Clostridium perfringens less abundant. |
| Aarnoutse et al. (2022a)/Netherlands | To explore the associations between intestinal microbiota, chemotherapy toxicity, and treatment response in postmenopausal estrogen receptor-positive (ER+) BC patients undergoing (neo)adjuvant chemotherapy. | 44 postmenopausal ER+ breast cancer patients. 18 patients received neoadjuvant chemotherapy (before surgery). 26 patients received adjuvant chemotherapy (after surgery). | During chemotherapy, species richness and microbial diversity significantly declined, with the lowest diversity one month post-treatment; Proteobacteria and unclassified Enterobacterales increased, while beneficial bacteria like Ruminococcaceae, Christensenellaceae, and Marvinbryantia decreased, with Enterobacterales and Lactobacillus rising during AC-D treatment but falling afterward, and Ruminococcaceae and Christensenellaceae recovering post-treatment. |
| Shoji et al. (2021)/ Japan | To evaluate the gut microbiome profile in obese patients with CRC and its relationship with metabolic markers. | 36 patients with CRC. 38 control participants without CRC. | CRC patients had higher microbial diversity compared to controls. Beta diversity significantly differed between CRC patients and controls. Obese CRC patients had significantly lower Enterococcus abundance compared to non-obese CRC patients. Capnocytophaga and Polaribacter were significantly more abundant in obese CRC patients. |
| Avelar-Barragan et al. (2023)/ Not mentioned | To characterize the gut microbiome of patients with myeloproliferative neoplasms (MPN) undergoing a Mediterranean diet intervention and assess its impact on inflammation and disease progression. | 28 individuals diagnosed with Philadelphia chromosome-negative MPNs (including Polycythemia Vera (PV), Essential Thrombocythemia (ET), and Myelofibrosis (MF)) and divided into mediterranean diet and USDA diet cohorts. | Increased inflammation markers (such as TNFα and IL-12p70) correlated with reduced microbial species richness, particularly in MF patients. Notable significant correlations included associations with TNFα vs *Flavonfractor*, IL-12p70 vs *Roseburia*, and IL-8 vs *Eubacterium*. |
| Uchino et al. (2021)/Japan | To evaluate the role of oral microbiota in CRC progression and investigate its potential as a biomarker for CRC detection. | 52 colorectal cancer patients and 51 healthy controls, aged 40 years or older. | Four bacterial species (*Peptostreptococcus stomatis, Streptococcus anginosus, Solobacterium moorei,* and *Streptococcus koreensis*) were found in significantly higher abundance in both the saliva and stool samples of CRC patients compared to healthy controls. *Solobacterium moorei* had a higher relative abundance in advanced-stage CRC patients (Stage III & IV) compared to early-stage CRC patients (Stage I & II). |
| Frugé et al. (2018)/USA | To explore the relationships between diet, gut microbiota, and Gleason sum in overweight and obese prostate cancer patients enrolled in a presurgical weight loss trial. | 40 overweight and obese prostate cancer patients in the southeastern. | At baseline, *Proteobacteria* were abundant, with four orders positively associated with Gleason sum. Increased red meat consumption was associated with lower *Prevotella* abundance and higher *Blautia* abundance. Increased poultry intake was associated with decreased *Clostridiales* abundance. |
| Hoang et al. (2023)/South Korea | To investigate how lifestyle factors (smoking, alcohol consumption), metabolic diseases (obesity, hypertension, diabetes), and dietary diversity influence gut microbiome composition and variation in CRC patients. | 331 colorectal cancer patients who underwent resection surgery | Dietary diversity showed no significant link to microbiome variation, suggesting limited dietary impact on microbiome composition; obese patients had more Faecalibacterium prausnitzii, Blautia obeum, and Sutterella, while non-obese had more Fusobacterium and Ruminococcus albus, with obesity and hypertension linked to a more stable, homogeneous microbiome; smokers had higher Streptococcus and Hafnia alvei, non-smokers had more Bilophila and Synergistes, alcohol consumers had increased Enterococcus and Citrobacter, and non-drinkers had higher Rothia. |
| Xie et al. (2019)/China | To investigate the effects of prebiotics (containing fructooligosaccharides, xylooligosaccharides, polydextrose, and resistant dextrin) on immune function and intestinal microbiota structure in perioperative CRC patients. | Patients were randomly divided into two groups: Prebiotic group (n = 70): Received prebiotic supplementation of 30 g/day for 7 days. Control group (n = 70): Did not receive prebiotic supplementation. | Prebiotic supplementation increased Bifidobacterium and Enterococcus preoperatively while reducing Bacteroides, which remained low post-surgery; in the control group, Enterococcus, Bacillus, Lactococcus, and Streptococcus increased post-surgery, but the prebiotic group only saw a rise in Escherichia-Shigella, with surgical stress increasing opportunistic pathogens and commensal bacteria in both groups, though moderated in the prebiotic group. |
| Wada et al. (2010)/Japan | To evaluate the effects of enteral administration of *Bifidobacterium breve* strain Yakult on infection prevention, fecal microbiota composition, and intestinal environment in pediatric cancer patients undergoing chemotherapy. | 42 pediatric patients with malignancies admitted for chemotherapy. Patients were randomized into two groups: Probiotic group (n = 19) and Placebo group (n = 23). | The probiotic group maintained higher levels of anaerobes, including *Clostridium leptum* subgroup. The placebo group showed a greater increase in *Enterobacteriaceae* (p = 0.03), an opportunistic pathogen. |
| Bobin-Dubigeon et al. (2021)/ France | To characterize and compare the fecal microbiota composition of early-stage BC patients and healthy women, identifying potential microbiome differences associated with BC. | 25 BC patients (before any therapeutic treatment) and 30 healthy controls. | Breast cancer patients had lower microbial diversity, with a reduced Shannon index, enriched Firmicutes, and decreased Bacteroidetes compared to healthy controls; Clostridium clusters IV and XIVa and Blautia were more abundant, while Bifidobacterium, Odoribacter, Butyricimonas, and Coprococcus were less abundant in BC patients. |
| Frugé et al. (2020)/USA | To investigate the association between fecal *Akkermansia muciniphila* (AM) abundance, body composition, gut microbiota diversity, and diet in overweight and obese women with early-stage BC participating in a presurgical weight-loss trial. | 32 overweight and obese women with early-stage (stage 0-II) breast cancer, enrolled in a presurgical weight-loss trial. | *A. muciniphila* had a bimodal distribution in the study population, with participants classified into low AM (LAM) and high AM (HAM) groups based on median AM relative abundance. HAM participants had higher alpha diversity at baseline than LAM. Change in dietary fiber intake was positively associated with *A. muciniphila* abundance in the LAM group. Higher intake of polyunsaturated fatty acids (PUFAs) and protein was also linked to increased microbiota diversity. |
| Amitay et al. (2017)/ Germany | To examine the prevalence and relative abundance of *Fusobacterium* in stool samples from participants at various stages of CRC development. | 500 participants from the BliTz screening colonoscopy study, including. | *Fusobacterium* was significantly more abundant in CRC patients than in all other groups. *Fusobacterium* was positively associated with more advanced CRC stages. No significant association was found between *Fusobacterium* abundance and dietary or lifestyle factors. |
| Smith et al. (2021)/USA | To compare the gut microbiome of newly diagnosed, treatment-naïve overweight and obese breast and prostate cancer patients with matched cancer-free controls to identify microbial differences associated with cancer. | 44 participants, including: 22 cancer patients (14 breast cancer, 8 prostate cancer) from presurgical weight-loss trials. 22 cancer-free controls. | Prostate cancer patients showed higher alpha diversity and distinct beta diversity compared to cancer-free males, with increased Tissierellaceae, Lachnospiraceae, and Ruminococcaceae, while healthy controls had more Veillonellaceae, suggesting a cancer-protective role; most genera were more abundant in controls, except Allobaculum, which was higher in breast cancer patients. |
| Haberman et al. (2023)/ Israel | To identify gut microbial signatures associated with lung cancer (LC) and determine specific bacterial taxa linked to durable clinical benefit (DCB) in advanced LC patients receiving treatment, particularly immune checkpoint inhibitors (CPIs). | 106 participants, including: 75 lung cancer patients (50 treated with CPIs, 25 not treated with CPIs) and 31 matched healthy volunteers | LC patients had lower microbial alpha diversity compared to controls. Beta diversity was significantly different between LC patients and controls. LC patients had lower abundance of beneficial taxa, including *Clostridiales, Lachnospiraceae*, and *Faecalibacterium prausnitzii*. Higher *Akkermansia muciniphila* abundance correlated with better DCB. *Clostridium citroniae* was enriched in patients with poor prognosis. |
| Marfil-Sanchez et al. (2021)/ Hungary and Austria | To evaluate the associations between gut microbiota and outcomes in lung cancer patients who underwent lung resection surgery. | 15 early-stage lung cancer patients who underwent lung resection surgery. | Post-surgery, Alistipes and Bacteroides species, linked to short-chain fatty acid production, increased, with more microbial correlations shifting from negative to positive (47) than positive to negative (37) across Actinobacteria, Bacteroidetes, Firmicutes, and Proteobacteria, notably involving Gemella sanguinis, Adlercreutzia equolifaciens, Lachnospiraceae bacterium 5 1 57FAA, Parabacteroides merdae, K. pneumoniae, and Barnesiella intestinihominis. |
| Allali et al. (2018)/Morrocco | To compare the stool microbiome composition of Moroccan CRC patients with healthy individuals to identify bacterial taxa relevant to the Moroccan population | 11 CRC patients and 12 healthy individuals​. | In CRC patients, Fusobacteria, Firmicutes, and Proteobacteria were overrepresented, while Prevotella was more abundant in controls and Bacteroides showed non-significant overrepresentation in CRC; significantly overrepresented species in CRC included Collinsella aerofaciens, [Eubacterium] biforme, Oxalobacter formigenes, Akkermansia muciniphila, and Bacteroides fragilis. |
| Nguyen et al. (2024)/ Vietnam | To profile the gut microbiome of Vietnamese patients with breast cancer and evaluate associations between gut microbial diversity, taxa abundance, and the gut microbiome health index (GMHI) with sociodemographic, clinical factors, and tumor characteristics | 356 Vietnamese patients with BC. | Post-surgery stool samples showed significantly lower alpha and beta diversity than pre-surgery samples. The mean GMHI was –0.79 pre-surgery and –2.81 post-surgery. Diagnosis delay was significantly associated with lower alpha diversity and variations in beta diversity. It was also linked to an increased abundance of *Enorma massiliensis* and decreased abundance of *Faecalicoccus pleomorphus*. High fiber intake was associated with lower alpha diversity and an increased abundance of *Bifidobacterium, Prevotella,* and *Bacteroides* species. |
| Guo et al. (2024)/China | To investigate gut microbiota and serum metabolite signatures along the colorectal adenoma-carcinoma sequence | Participants comprised 26 colorectal adenoma (CRA) patients, 19 CRC patients, 10 familial adenomatous polyposis (FAP) patients and 20 healthy controls. | Enriched in CRC patients: *Enterococcus*, *Pseudomonas*, *Fusobacterium*, *Alistipes*, *Blautia.* A combination of 5 microbial and 5 metabolite biomarkers effectively differentiated CRA from CRC with an AUC of 0.85. |
| Shi et al. (2024)/ China | To characterize the gut microbiota of CRC patients in Hubei, China and compare their microbial composition with cohorts from other regions in China.​ | Participants comprised 25 CRC patients and 26 healthy controls. | CRC patients exhibited a lower microbial diversity and enrichment of potentially pathogenic bacteria. Actinobacteriota, Desulfobacterota, and Proteobacteria were significantly more abundant in the CRC group. At the family level, Corynebacteriaceae, Enterobacteriaceae, Enterococcaceae, and Lactobacillaceae were significantly more abundant in the CRC group. CRC patients had an increased abundance of Bilophila, Corynebacterium, Enterococcus, Lactobacillus, Clostridium]_innocuum_group and Escherichia-Shigella after p-value correction. |
| Pellegrini et al. (2020)/Italy | To evaluate the effects of combining Bifidobacterium longum BB536 and Lactobacillus rhamnosus HN001 probiotics with a Mediterranean diet (MD) versus MD alone on gut microbiota, body weight, and metabolic and inflammatory serum markers in overweight breast cancer survivors. | Participants included 34 overweight female breast cancer survivors, cancer-free at the time of enrollment. | After 2 months of probiotics, the number of bacterial species and bacterial diversity significantly increased in the intervention group, while no changes were observed in the control group. Probiotic supplementation influenced gut microbiota composition but had no significant effect on dietary adherence​. |
| Cai et al. (2023)/China | To investigate how gut microbiota enterotypes mediate the relationship between dietary patterns and colorectal neoplasm (CRN) risk. | Groups were 130 CRC patients, 120 CRA patients and 160 control participants. | Healthy dietary pattern was associated with lower CRC risk in individuals with Type I and Type II enterotypes. Type III enterotype (Prevotella 9-dominated) showed no significant association with diet and CRC risk. Type I CRC (Bacteroides-Lachnoclostridium) and Type II CRC (Bacteroides-Faecalibacterium) had higher short-chain fatty acid (SCFA)-producing bacteria. Type III CRC (Prevotella 9-dominated) had higher levels of trimethylamine (TMA)-producing bacteria. |
| Li et al. (2022)/ China | To analyze gut microbiota variations across different colorectal tissue sites, including tumor mucosa, para-cancerous mucosa, normal mucosa, and feces, in CRC patients. | Total sample size was 98 CRC patients. | Tumor mucosal microbiota was enriched with *Fusobacterium*, *Gemella*, and *Campylobacter*. Para-cancerous mucosa had microbial characteristics intermediate between tumor and normal mucosa. Fusobacterium abundance was strongly correlated with FOBT positivity. SCFA-producing bacteria (e.g., *Blautia*, *Roseburia*) were lower in mucosal samples compared to feces. |
| Zhou et al. (2022)/USA | To examine the differences in gut microbiome composition between children with solid tumors post-chemotherapy and healthy controls, and to investigate how macronutrient and antioxidant nutrient intake correlate with gut microbiome diversity | The study included 49 children (27 with solid tumors, 22 healthy controls) with a mean age of 14.4 years in the cancer group and 12.1 years in the control group. | Children with inadequate fat intake based on calories percentage had higher abundances in family S24-7 and in genus Megasphaera while children with adequate fat intake had higher abundances in bacterial family Erysipelotrichaceae and Peptostreptococcaceae. Beta carotene intake had a positive correlation with Faith’s_PD and a trend of positive association with Chao1. The amount of selenium intake was negatively correlated with Shannon’s index and Pielou’s_e. |
| Alhhazmi et al. (2023)/ Saudi Arabia | To analyze the gut microbiota composition and metabolic pathway enrichment in late-stage CRC patients in Saudi Arabia and identify microbial dysbiosis patterns associated with CRC progression | The study included 50 participants, with 25 CRC patients (mean age 54.32 ± 14.2 years) and 25 healthy controls (mean age 47.40 ± 11.72 years). | CRC patients had significantly altered gut microbiota, with lower diversity and distinct microbial signatures compared to healthy controls. Enriched bacterial genera in CRC patients included: *Streptococcus salivarius, Streptococcus parasanguinis, Streptococcus anginosus, Lactobacillus mucosae, Lactobacillus gasseri, Peptostreptococcus, Eubacterium, Aerococcus, Escherichia-Shigella, Klebsiella, Enterobacter, Alistipes, and Ralstonia.* |
| Xu et al. (2023)/China | To investigate how neo-adjuvant radiotherapy (NART) influences intestinal microbiota composition in rectal cancer patients | The study included five rectal cancer patients (28 stool samples collected) who received NART and five healthy family members (16 stool samples collected) as controls. | NART did not significantly alter microbiota alpha diversity but affected bacterial composition. Bacterial genera associated with cancer progression, such as *Enterobacter, Citrobacter, Peptoniphilus, Dialister, and Intestinibacter*, decreased after prolonged NART. |
| Aarnoutse et al. (2021)/Netherlands | To investigate differences in intestinal microbiota between postmenopausal breast cancer patients and healthy controls and assess the impact of systemic cancer treatment on gut microbiota composition. | The study included 148 postmenopausal women, with 81 BC patients and 67 controls. | Observed species richness and the Shannon index were not different between breast cancer patients and the controls. breast cancer stage was negatively correlated to the abundance of Veillonellaceae and Dialister. In line with this, increasing clinical tumor size was associated with a lower abundance of Veillonellaceae. Other clinical characteristics showed no significant correlations with differentially abundant taxa. |
| Serrano et al. (2021)/Italy | The study aimed to evaluate the role of the gut microbiome as a mediator in the relationship between diet and CRC risk, considering the influence of vitamin D, inflammatory markers, and adipokines. | The study included 84 participants recruited at the European Institute of Oncology, consisting of 34 CRC patients and 32 controls. | Data showed that cases had a significantly higher abundance of Escherichia coli, Parvimonas micra, and Solobacterium moorei species. In CRC patients, the study found significant associations with Parvimonas micra, Fusobacterium nucleatum, and Bacteroides fragilis  species. Researchers also found a significantly greater percentage of CRC patients with low levels of 25-OHD had a high abundance of Parvimonas genus. |
| Richard et al. (2018)/ France and Italy | To investigate the differences in mucosa-associated microbiota composition between colitis-associated cancer (CAC), sporadic colorectal cancer (SC), and healthy subjects (HS). | The study included 7 CAC patients, 10 SC patients, and 10 healthy subjects. | CAC patients exhibited distinct bacterial dysbiosis compared to SC and healthy subjects, characterized by increased Enterobacteriaceae (including Escherichia-Shigella) and Sphingomonas and a depletion of Fusobacterium and Ruminococcus. SC patients had a strong increase in Fusobacterium, while CAC patients showed a marked enrichment in Proteobacteria. Alpha diversity was significantly reduced in CAC. Streptococcus species were significantly enriched in CAC tumor sites. |
| Bai et al. (2024)/USA | To investigate the associations between the gut microbiome, metabolome, and psychoneurological symptoms (PNS) in children with cancer receiving chemotherapy, using a multi-omics approach to identify microbiome–metabolome pathways linked with PNS development​ | The study included 35 children (21 with cancer, 14 healthy controls), with a mean age of 13.2 years in cancer cases and 13.1 years in controls. | Alpha diversity was significantly lower in cancer patients (both T0 and T1) compared to healthy controls, with further microbial depletion post-chemotherapy. Cancer patients at T0 had higher levels of *Ruminococcus, Megasphaera, and Prevotella*. At T1, *Megasphaera* remained dominant and was linked to aspartate/asparagine metabolism, carnitine shuttle, and tryptophan metabolism. PNS were negatively associated with gut microbes (e.g., *Lactobacillus*, *Bifidobacterium,* and *Roseburia*) at T0. |
| Hoang et al. (2022)/South Korea | The study aimed to investigate the association between diet and the overall diversity and different taxa levels of the gut microbiota in CRC patients using a nutrition-wide association approach. | The study included 115 CRC patients who underwent CRC surgery at the Department of Surgery, Seoul National University Hospital. | Negative correlations were observed between Bacteroides fragilis and the intake of pork belly, beef soup with vegetables, animal fat, and fatty acids. Clostridium symbiosum showed inverse correlations with the intake of some fatty acids, amines, and amino acids. High intake of seaweed was associated with a 6% lower abundance of Rikenellaceae and a 7% lower abundance of Alistipes. |
| Juan et al. (2021)/China | The study aimed to evaluate whether probiotics could reduce docetaxel-related weight gain in breast cancer patients undergoing chemotherapy. | The study included 100 female BC patients (Stage I-III) receiving docetaxel-based chemotherapy. | After docetaxel-based chemotherapy, the relative abundance of Tenericutes on phylum level in the probiotics group was signficantly higher than that in the placebo group. There were higher levels of [Eubacterium]_coprostanoligenes_group, and lower levels of Bacteroides, and Anaerostipes on the genus level in the probiotics. |
| Gao et al. (2015)/ China | The study aimed to assess whether perioperative oral probiotics could alter the microbial composition and improve gut microecology in CRC patients. | The study included 22 CRC patients undergoing radical colectomy at Shanghai Jiao Tong University Affiliated Sixth People's Hospital. | Patients receiving probiotics had significantly higher Chao and ACE indices. CRC patients had lower microbial diversity than healthy controls, but probiotic supplementation increased gut microbiota diversity closer to normal levels. *Fusobacterium* abundance, which was significantly higher in CRC patients, was reduced by ~6-fold in the probiotic group. *Peptostreptococcus* and *Comamonas* were also significantly reduced in the probiotic group. The probiotic group showed a higher relative abundance of Enterococcus and Proteobacteria. |
| Dizman et al. (2021)/USA | The study aimed to assess whether a Bifidobacterium-containing yogurt supplement could modulate the gut microbiome and improve clinical outcomes in patients with metastatic renal cell carcinoma (mRCC). | A total of 20 patients with metastatic renal cell carcinoma (mRCC) were enrolled. | *Bifidobacterium animalis* was detected in 67% of post-baseline stool samples in the probiotic group but only 0.023% in the probiotic-restricted group. Patients who achieved clinical benefit (partial response or stable disease for over 6 months) had significantly higher levels of Barnesiella intestinihominis and Akkermansia muciniphila. Clinical benefit rate was similar between probiotic-supplemented and probiotic-restricted arms. |
| Sánchez-Alcoholado et al. (2021)/ Spain | The study aimed to assess the relationship between gut microbiota composition, microbial-derived metabolites (short-chain fatty acids and polyamines), and the pathological response to neoadjuvant radiochemotherapy (RCT) in CRC patients. | The study included 40 CRC patients (stages II–III, T2–T4, and/or N1–N2) undergoing neoadjuvant RCT. | Responders (R) to treatment showed higher microbial diversity, increased beneficial bacteria (Bifidobacterium bifidum, Ruminococcus albus, Roseburia, Faecalibacterium prausnitzii), and decreased pro-inflammatory bacteria (Fusobacterium nucleatum, Bacteroides fragilis, Escherichia coli, Prevotella copri, Klebsiella) compared to non-responders (NR); Prevotella copri was linked to higher zonulin and intestinal permeability, while post-treatment saw increased Bifidobacterium and decreased Fusobacterium, Escherichia, and Klebsiella; Firmicutes and Bacteroidetes remained stable, but Actinobacteria and Firmicutes increased, and Fusobacteria and Proteobacteria decreased in R versus NR at T3, with Ruminococcaceae and Bifidobacteriaceae more abundant and Prevotellaceae, Enterobacteriaceae, and Fusobacteriaceae less abundant in R. |
| Hong et al. (2024)/China | The study aimed to evaluate the effects of a sterile diet versus a normal diet on gut microbiome dynamics in leukemia patients post-hematopoietic cell transplantation (HCT). | The study followed nine leukemia patients (three males, six females, median age: 13 years, range: 3–34 years) who underwent umbilical cord blood transplantation (UCBT). A control group of nine healthy family members was included for comparison. | Sterile diet caused gut microbiome collapse: Significant reduction in microbiota diversity (p < 0.05) during the sterile diet, 85.33% reduction in microbial interaction network edges. Microbial richness and interactions recovered after transitioning to a normal diet: Beneficial bacteria increased (*Bifidobacterium, Blautia, Romboutsia*) and Pathogenic bacteria decreased (*Streptococcus, Enterococcus, Lactobacillus*). The gut microbial network became less connected and weaker during the sterile diet. A normal diet increased microbial cohesion and stability, restoring network complexity. |
| Kim et al. (2024)/ South Korea | The study aimed to evaluate the impact of a modified microbiota-accessible carbohydrate (mMAC) diet on gut microbiota composition and clinical symptoms, including bowel habits, in CRC patients after surgical resection. | The study included 40 colorectal cancer patients (Stages I–III, median age: 61 years, range: 38–82 years) who had undergone curative surgical resection. | Taxonomic shifts occurred, including increased Lactobacillus, Bifidobacterium, and Prevotella, particularly in the non-chemotherapy group. After the mMAC diet, Prevotella abundance increased, which correlated positively with acetate and propionate production. In chemotherapy patients, Fusobacterium and Catabacter decreased after the mMAC diet, while Lactococcus increased. Stage III CRC patients had higher levels of Rothia, Coprobacillus, and Veillonella, while left-sided CRC patients had higher Sellimonas and Eubacterium. |
| Yuan et al. (2024)/China | The study aimed to evaluate the characteristics of intestinal flora, nutritional status, and immune function in obese colon cancer patients with different histological subtypes. | The study conducted a retrospective analysis of 64 obese colon cancer patients diagnosed between June 2018 and January 2020. | Bifidobacterium, Lactobacillus and Enterococcus levels significantly decreased after surgery. Escherichia coli and yeast increased significantly postoperatively, suggesting potential dysbiosis. No significant differences in microbiota composition were observed among the different histological subtypes pre- and post-surgery. |
| Aarnoutse et al. (2022b)/ Netherlands | The study aimed to investigate the association between intestinal microbiota composition and treatment response in metastatic colorectal cancer (mCRC) patients receiving capecitabine. | The study included 33 mCRC patients receiving capecitabine, with or without bevacizumab, across four Dutch hospitals. | Beta-diversity showed large inter-individual variability, with no significant clustering of responders versus non-responders. Capecitabine treatment did not significantly alter microbiota composition. One patient with high baseline Bifidobacterium levels had the greatest tumor response (40% reduction in target lesions), suggesting a potential link between Bifidobacterium and treatment efficacy. |
| Cao et al. (2022)/ China | To evaluate the effects of oral administration of Clostridium butyricum on inflammation, immunity, intestinal microbiota, and postoperative recovery in gastric cancer patients following gastrectomy. | The study included 92 gastric cancer patients who underwent gastrectomy, divided into two groups: the AGP group (n=45, treated with placebo) and the AGA group (n=47, treated with C. butyricum). | *C. butyricum* supplementation reduced postoperative inflammation, enhanced immunity, and improved recovery. The AGA group had higher albumin levels, increased gut microbial diversity, distinct microbial composition, higher intestinal SCFAs, fewer gastrointestinal adverse reactions, and reduced complications compared to the AGP group. C. butyricum greatly enriched the relative abundance of beneficial bacteria Bacteroides, Faecalibacterium and Gemmiger, while the abundance of pathogenic Streptococcus, Desulfovibrio and Actinomyces were markedly decreased at genus level. |
| Donati Zeppa et al. (2023)/ Italy | To investigate the impact of a 12-week home-based lifestyle intervention, combining MD adherence and aerobic exercise, on gut microbiota composition and its association with cardiometabolic parameters in BC survivors during the first wave of the COVID-19 lockdown. | The study included 20 female breast cancer survivors with a mean age of 51.8 ± 7.8 years. Participants were 10.2 ± 3.1 months post-diagnosis, with disease stages ranging from 0 (20%) to I (50%) and II (30%). | The 12-week lifestyle intervention significantly increased microbial richness. At the phylum level, Proteobacteria decreased significantly. At the order level, significant increases occurred in Lactobacillales, Acidaminococcales, and Burkholderiales, though not significant after FDR correction. At the genus level, significant decreases were observed in Odoribacter, Erysipelotrichaceae_UCG-003, Coprococcus, Lachnospiraceae_UCG-004, and Sutterella, with an increase in Colidextribacter. Correlations showed negative associations between fasting glucose and Clostridia_vadinBB60, insulin and HOMA index with Butyricicoccus, and HDL cholesterol with Escherichia/Shigella, while MD adherence positively correlated with Lachnospiraceae_ND3007, Faecalibacterium, and Butyricimonas. |
| Li et al. (2025)/China | To evaluate the effects of oral nutritional supplementation (ONS) combined with probiotics on liver function and intestinal microflora in lung cancer patients undergoing chemotherapy. | The study involved 113 lung cancer patients receiving chemotherapy. | The amount of *Lactobacillus acidophilus*, *Bifidobacterium longum*, and *Bacteroides fragilis* in the intervention group were significantly increased than that in the control group, while the amount of *Escherichia coli* and *Enterococcus facalis* were significantly decreased after intervention. |
| Sitthideatphaiboon et al. (2024)/Thailand | To investigate the association between dietary patterns and the gut microbiome in Thai patients with advanced non-small cell lung cancer (NSCLC) undergoing immune checkpoint inhibitor (ICI) therapy | The study included 57 Thai patients with advanced NSCLC receiving ICI therapy, with a mean age of 60 years (SD 7.55–7.71). | Alpha diversity (Shannon indices) was significantly higher in the HPD group compared to the non-HPD group. Specific taxa enriched in the HPD group included the Candidatus Clisobacter genus and Alphaproteobacteria family, while the non-HPD group showed enrichment of Pasteurellales order, Pasteurellaceae family, and genera such as Anaerostipes, Veillonella, Meliiternanchacter, and Rikenella. Species-level analysis identified Anaerostipes, Veillonella, and Lactococcus lactis as enriched in the non-HPD group, while Desulfovibrio was more abundant in the non-HPD group. |
